# Natural History Study of Retinal Structure, Progression, and Symmetry Using Ellipzoid Zone Metrics in *RPGR*-Associated Retinopathy

**DOI:** 10.1016/j.ajo.2018.10.003

**Published:** 2019-02

**Authors:** James J.L. Tee, Yesa Yang, Angelos Kalitzeos, Andrew Webster, James Bainbridge, Michel Michaelides

**Affiliations:** University College London Institute of Ophthalmology and Moorfields Eye Hospital, London, United Kingdom

## Abstract

**Purpose:**

This is a quantitative study of retinal structure, progression rates, and interocular symmetry in retinitis pigmentosa GTPase regulator gene (*RPGR*)-associated retinopathy using spectral-domain optical coherence tomography (OCT).

**Design:**

Prospective, observational cohort study.

**Methods:**

Thirty-eight subjects at Moorfields Eye Hospital in London were assessed with 2 spectral-domain OCT–derived ellipzoid zone (EZ) metrics with repeatability assessments. EZ width (EZW) measurements were made on transfoveal line scans. En face images of the EZ area (EZA) were generated from high-density macular volume scans and were quantified. Baseline size, progression rate, symmetry, associations with age and genotype, and baseline structure–function correlation were investigated.

**Results:**

Baseline EZW and EZA measurements were 1963.6 μm and 3.70 mm^2^, respectively. The mean EZW progression rate was 233.6 μm per year, and the mean EZA rate was 0.67 mm^2^ per year. Relative interocular difference as an index of symmetry was 3% for both metrics, indicating good baseline symmetry in general—although significant variation existed across the cohort. Analysis of variance found a significant effect of age but not genotype on EZ dimension and progression rates. Larger EZ dimension and greater progression were seen in younger subjects. A positive correlation between EZ dimension and progression was evident. Overall exponential decline rates of 8.2% with EZW and 15.5% with EZA were obtained. Good functional correlation was found with EZW demonstrating stronger correlation; however, EZA correlation with function was also significant.

**Conclusions:**

EZ metrics are sensitive structural biomarkers for measuring residual extent and progression in *RPGR*-associated retinopathy. Our elucidation of the natural history will provide clinicians and patients with more knowledge about the condition and inform the design and interpretation of interventional trials.

Retinitis pigmentosa (RP) caused by sequence variants in the retinitis pigmentosa GTPase regulator gene (*RPGR*) constitute around three-quarters of all X-linked (XL) RP,^1^, ^2^, ^3^, ^4^ with *RP2* variants predominantly accounting for the remaining cases.^1^, ^3^, ^5^, ^6^, ^7^ There is particular interest in *RPGR*-associated RP, and recently commenced gene therapy trials are underway (NCT03116113, NCT03252847, and NCT03316560).

Spectral-domain optical coherence tomography (OCT) is in widespread use as an imaging modality to study retinal structure in a myriad of diseases. Previous OCT studies in RP have reported structural changes occurring at the transition zone as the scanned region of interest traverses from healthy central retinal tissue to diseased periphery.^8^, ^9^ Structural measurements of ellipzoid zone (EZ) width in RP, as a metric of disease severity and progression, have been shown to correlate well with retinal function.^10^, ^11^, ^12^, ^13^ Serial measurements of EZ width (EZW) to assess progression in XLRP have been studied^14^, ^15^, ^16^, ^17^; however, these studies were potentially limited by bias in eye selection,^14^, ^15^, ^16^ do not distinguish between genetic causes of XLRP,^14^, ^15^ or are retrospective.^17^ The quantification of ellipzoid zone area (EZA), made possible with the use of vendor software to construct en face images from spectral-domain OCT volume scans, has been shown to be feasible in quantifying progression in autosomal recessive (AR) RP.^18^ Despite this, there have not been further studies on the use of EZA as a structural metric in RP.

We therefore investigated the following in this protocol-driven prospective spectral-domain OCT study comprised solely of RP subjects with molecularly confirmed pathogenic *RPGR* mutations: (1) intraobserver repeatability with EZW and EZA metrics; (2) characterize baseline retinal structure with both metrics; (3) characterize progression with both metrics; (4) characterize interocular symmetry at baseline and progression and establish indices to quantify symmetry with both metrics; (5) investigate correlations between baseline measurements, progression, and age; (6) investigate effects of age and genotype on baseline and progression; (7) determine overall exponential rates of progression with both metrics; and (8) investigate structure–function correlations at baseline with both EZW and EZA metrics.

## Methods

Approval was granted by the ethics committee at Moorfields Eye Hospital before conducting the study. The Declaration of Helsinki was adhered to throughout. All subjects were affected males with RP caused by molecularly confirmed disease-causing mutations in *RPGR*. Bidirectional sequencing to test for mutations in *RPGR* exons 1 to 14 and open reading frame 15 (ORF15) was performed at the Central Manchester University Hospitals Genomic Diagnostics Laboratory (Manchester, UK) before recruitment.

### OCT Acquisition and Analysis

Dedicated clinical research ophthalmic technicians acquired protocol-driven OCT scans from both eyes of each subject at each visit, with the Spectralis OCT device (Heidelberg Engineering, Heidelberg, Germany). Horizontal high-resolution transfoveal line scans were obtained using automated real-time tracking (ART) with an average of 100 images. Automatic registration was used for all follow-up scans to ensure accurate correspondence of retinal locations. After line scan acquisition, bilateral macular high-resolution volume scans were acquired at the same visit. Each volume scan was comprised of 193 horizontal b-scans in high resolution mode with 30 μm distance between b-scans. Volume scans were purposefully acquired to allow the creation of en face images of the ellipzoid zone area (EZA). After a departmental upgrade to the Spectralis OCT imaging platform with OCT2 module (Heidelberg Engineering), volume scans acquired after June 2016 were captured with an average of 12 images (ART12) per horizontal b-scan, as allowed for by increased hardware capabilities and faster scanning speed with significant reduction in acquisition time.

**Figure 1:**
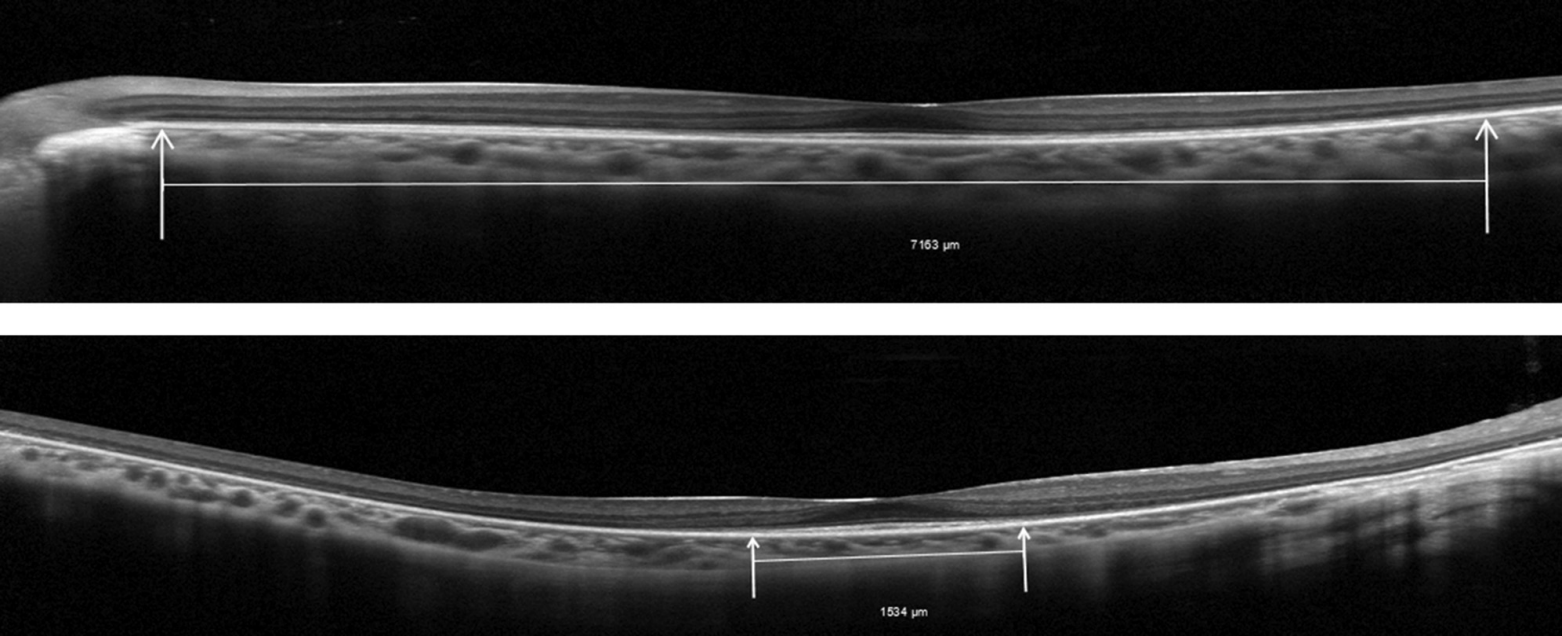
Horizontal transfoveal spectral domain optical coherence tomography scans of both eyes of a subject with retinitis pigmentosa GTPase regulator gene–associated retinopathy. Vertical arrows mark the extent of the ellipzoid zone on the scans. The ellipzoid zone width was 7163 μm in the left eye (top) and 1534 μm in the right eye (bottom). Note that the large interocular difference found in this subject is not in keeping with the cohort.

Image analyses for both EZW and EZA metrics were performed using vendor software (Heidelberg Eye Explorer version 1.10.2.0; Heidelberg Engineering). EZW analyses were conducted on transfoveal line scan images with the following methodology: images were displayed in a 1:1 μm setting. The nasal and temporal extents of the EZ (point of EZ disappearance into the proximal retinal pigment epithelium border with loss of outer segment layer) were identified and marked with the arrow tool. EZW was measured with the caliper tool as a straight line tangential to the distal retinal pigment epithelium border (Figure 1). Line scan images were analyzed in a random order for each subject.

**Figure 2:**
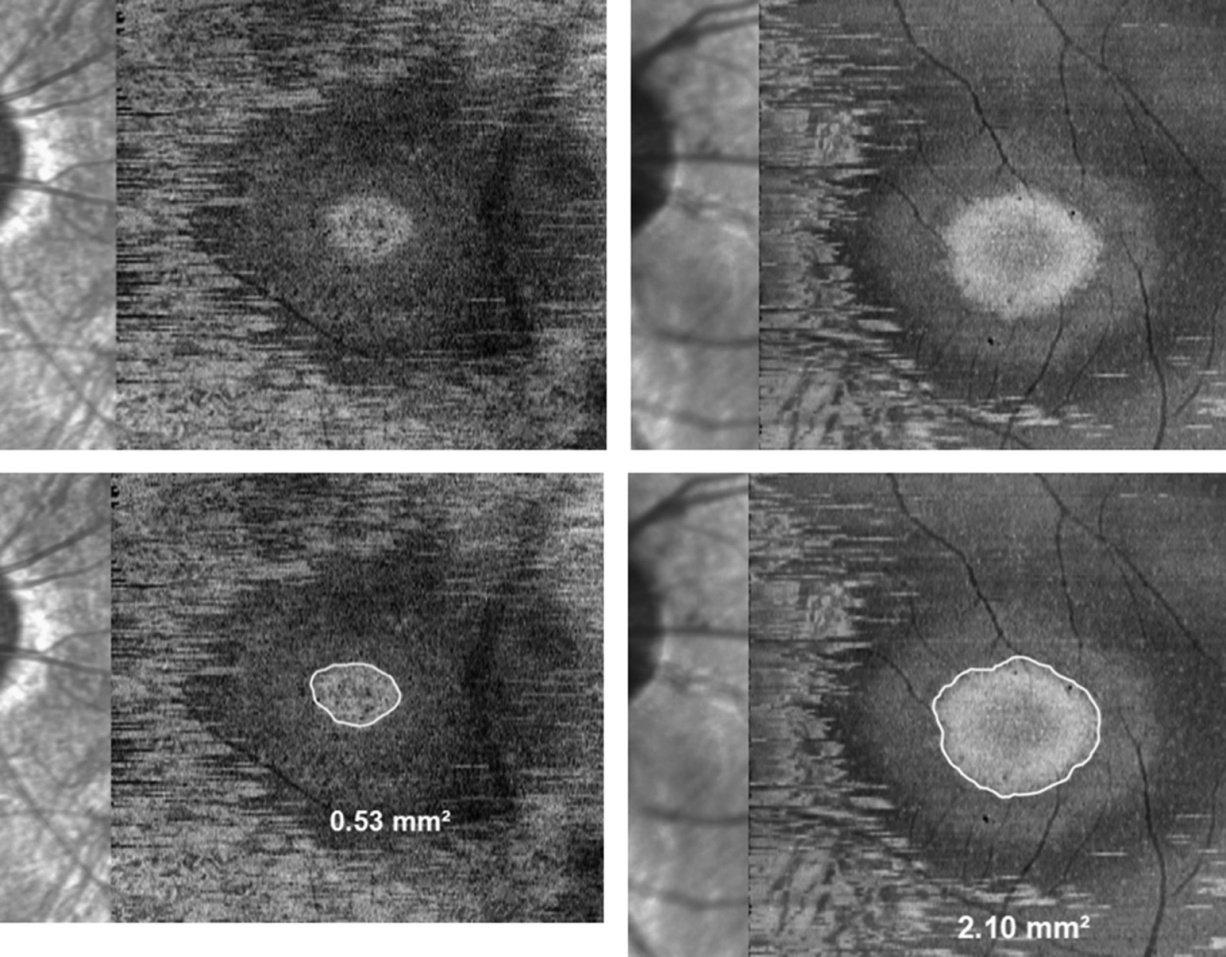
(Top) En face images of the ellipzoid zone area generated from high-resolution macular volume scans of 2 subjects with retinitis pigmentosa GTPase regulator gene–associated retinopathy. (Bottom) Delineation and quantification of respective ellipzoid zone areas from the top images. Images on the right column were generated from a macular volume scan acquired with an average of 12 images per b-scan.

En face images of the EZA were created from each macular volume scan with the following method using vendor software: images were created in a 3-dimensional mode and displayed in transverse view. The autosegmented Bruch membrane line was manually inspected for accurate placement along the outer border of the retinal pigment epithelium–Bruch membrane complex as this line was utilized as a reference for the slab contour. The reference line was displaced 25 μm inward from the Bruch membrane and a slab of 30 μm thickness was created. Slab settings were designed to capture the whole extent of the EZ layer in subjects. After image creation, the EZA was delineated and measured using minimum intensity projection with area value provided by the vendor software (Figure 2).

### Statistics

For the assessment of intraobserver repeatability, EZW and EZA baseline images of each eye were measured twice by a single observer, a minimum of 1 week apart. Intraobserver repeatability assessment was also conducted on EZA derived from ART12 images (EZA-ART12) acquired after the upgrade to OCT2. One image per eye per subject was used to maintain the “independence-of-score” and to avoid inducing systematic bias. The method popularized by Bland and Altman was used to calculate mean ± standard deviation (SD) of test–retest differences and corresponding 95% limits of agreement (LOA). Residuals were inspected for normality. Repeatability coefficient (1.96 × SD) and the test–retest variability for both metrics were calculated and these are shown in Table 1.

**Table 1:** Intraobserver Repeatability Analysis of EZW and EZA Measurements

Metric	EZW			EZA			EZA-ART12		
	Right Eyes	Left Eyes	Both Eyes	Right Eyes	Left Eyes	Both Eyes	Right Eyes	Left Eyes	Both Eyes
Mean ± SD of intraobserver difference (μm for EZW, mm^2^ for EZA)	−8.26 ± 121.55	34.32 ± 174.99	−21.29 ± 150.22	−0.062 ± 0.513	0.013 ± 0.551	−0.025 ± 0.529	−0.110 ± 0.228	−0.040 ± 0.404	−0.076 ± 0.326
95% LOA (μm for EZW/mm^2^ for EZA)	−246.49 to 229.97	−377.29 to 308.66	−315.72 to 273.14	−1.067 to 0.943	−1.067 to 1.093	−1.062 to 1.013	−0.561 to 0.341	−0.824 to 0.744	−0.727 to 0.567
Repeatability coefficient	238.24	342.98	294.43	1.005	1.080	1.037	0.4508	0.7840	0.6468
Test–retest variability, %	12.38	17.12	14.99	29.82	26.93	28.03	13.38	19.55	17.48

ART12 = automatic real-time tracking with average of 12 images per horizontal b-scan; EZA= ellipzoid zone area; EZW= ellipzoid zone width; LOA= limit of agreement; SD = standard deviation.

Test–retest measurements were performed a minimum of 1 week apart on 76 eyes of 38 subjects for the EZW metric, 62 eyes of 31 subjects for the EZA metric, and 53 eyes of 27 subjects for EZA-ART12. Repeatability coefficient = 1.96 (SD).

**Figure 3:**
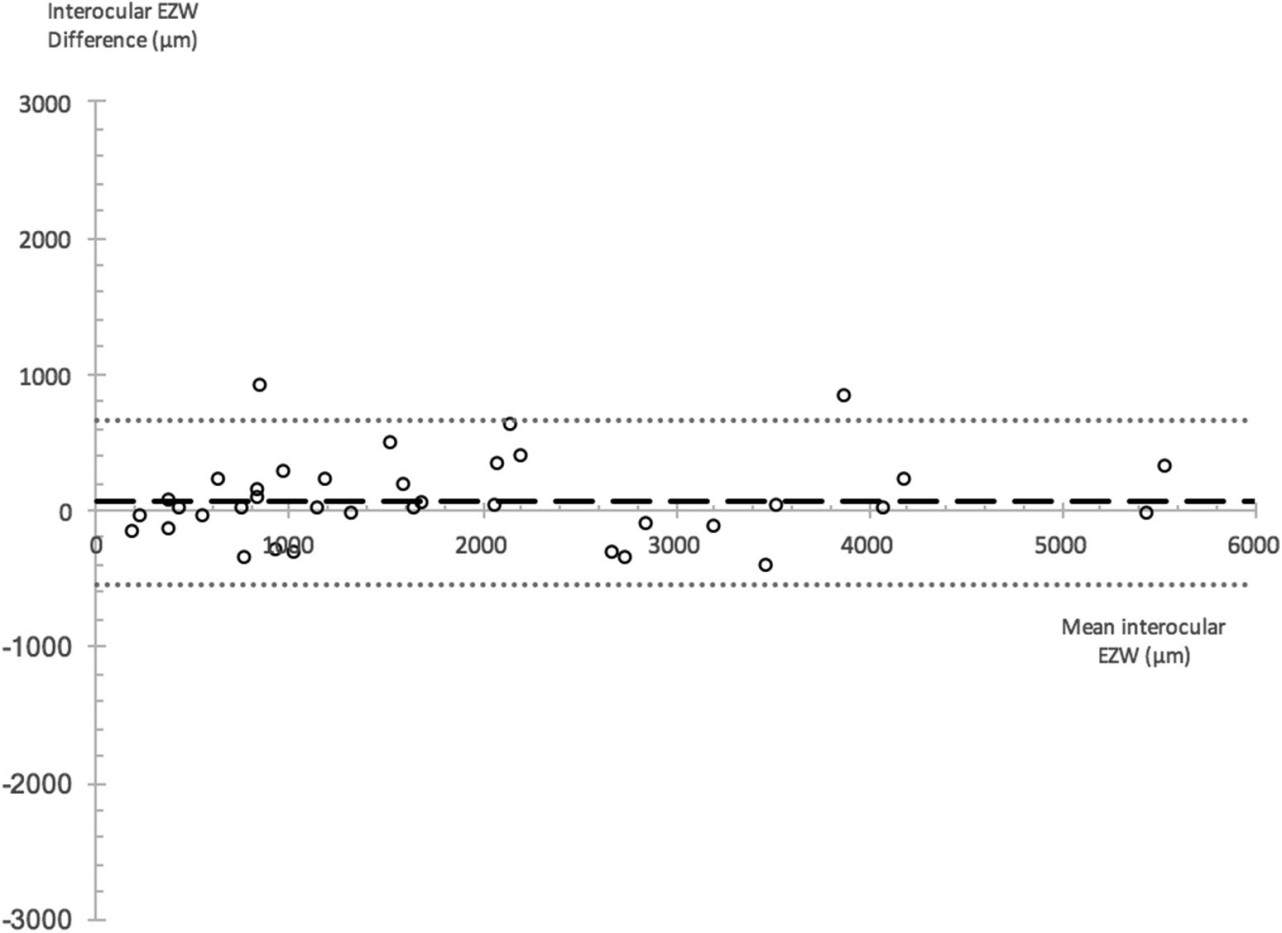
Bland-Altman plot showing interocular differences in ellipzoid zone width (EZW) at baseline. Interocular difference for each individual is plotted on the y axis against the mean EZW value of both eyes. The long horizontal dashed line refers to the mean of interocular differences; horizontal dotted lines denote 95% limits of agreement.

The Bland-Altman method was further used to assess interocular symmetry at baseline and progression as observed with both metrics (Table 2). An example is provided in Figure 3. Baseline EZW and EZA, EZW % rate, and EZA % rate residuals were inspected for normality. The mean ± SD of interocular difference, together with 95% LOA, was calculated. Interocular differences were obtained from the subtraction of left from right eyes. The mean of interocular differences was expressed as a fraction of its corresponding cohort average (the cohort average in turn was calculated by obtaining the average of all interocular [mean of right and left eye] values of subjects), and this was presented as the relative interocular difference (RID). The interocular coefficient (1.96 × SD), expressed as a fraction of the corresponding cohort average, was calculated and presented as the relative interocular variability (RIV). These indices were calculated to facilitate metric cross-comparison.

**Table 2:** Baseline Values and Progression Rates for EZW and EZA With Respective Indices of Interocular Symmetry

Metric	All Eyes		Right Eyes		Left Eyes		Indices of Interocular Symmetry				
	Mean (SD)	95% CI	Mean (SD)	95% CI	Mean (SD)	95% CI	Mean (SD) of Interocular Difference	95% LOA	RID, %	Interocular Coefficient	RIV, %
EZW baseline (μm)	1963.59 (1541.76)	1611.28–2315.90	1923.79 (1447.35)	1448.06–2399.52	2003.39 (1649.31)	1461.28–2545.51	63.51 (308.79)	−541.72 to 668.74	3.34	605.23	31.87
EZA baseline (mm^2^)	3.70 (6.14)	2.13–5.25	3.37 (5.60)	1.31–5.42	4.01 (6.72)	1.55–6.48	0.10 (0.30)	−0.49 to 0.70	3.07	0.59	17.58
EZW rate (μm/yr)	233.55 (189.13)	182.90–284.20	236.82 (192.20)	162.30–311.35	230.28 (189.49)	156.80–303.75					
EZW rate (%/yr)	13.20 (14.85)	9.22–17.18	13.59 (15.54)	7.57–19.62	12.81 (14.41)	7.22–18.39	0.20 (6.94)	−13.39 to 13.8	1.54	13.60	102.60
EZA rate (μm/yr)	0.67 (0.95)	0.38–0.97	0.61 (0.83)	0.24–0.99	0.73 (1.07)	0.24–1.21					
EZA rate (%/yr)	16.70 (11.19)	13.22–20.19	15.96 (9.56)	11.61–20.31	17.45 (12.81)	11.62–23.28	−1.49 (6.65)	−14.52 to 11.54	8.93	13.03	78.01

CI = confidence interval; EZA = ellipzoid zone area; EZW = ellipzoid zone width; LOA= limit of agreement; RID = relative interocular difference; RIV = relative interocular variability; SD = standard deviation.

The number of eyes in the cohort for characterization of baseline EZW were 76 eyes of 38 subjects, baseline EZA with 62 eyes of 31 subjects, EZW rate with 56 eyes of 28 subjects, and EZA rate with 42 eyes of 21 subjects. Positive progression rates indicate constriction occurring over time.

**Figure 4:**
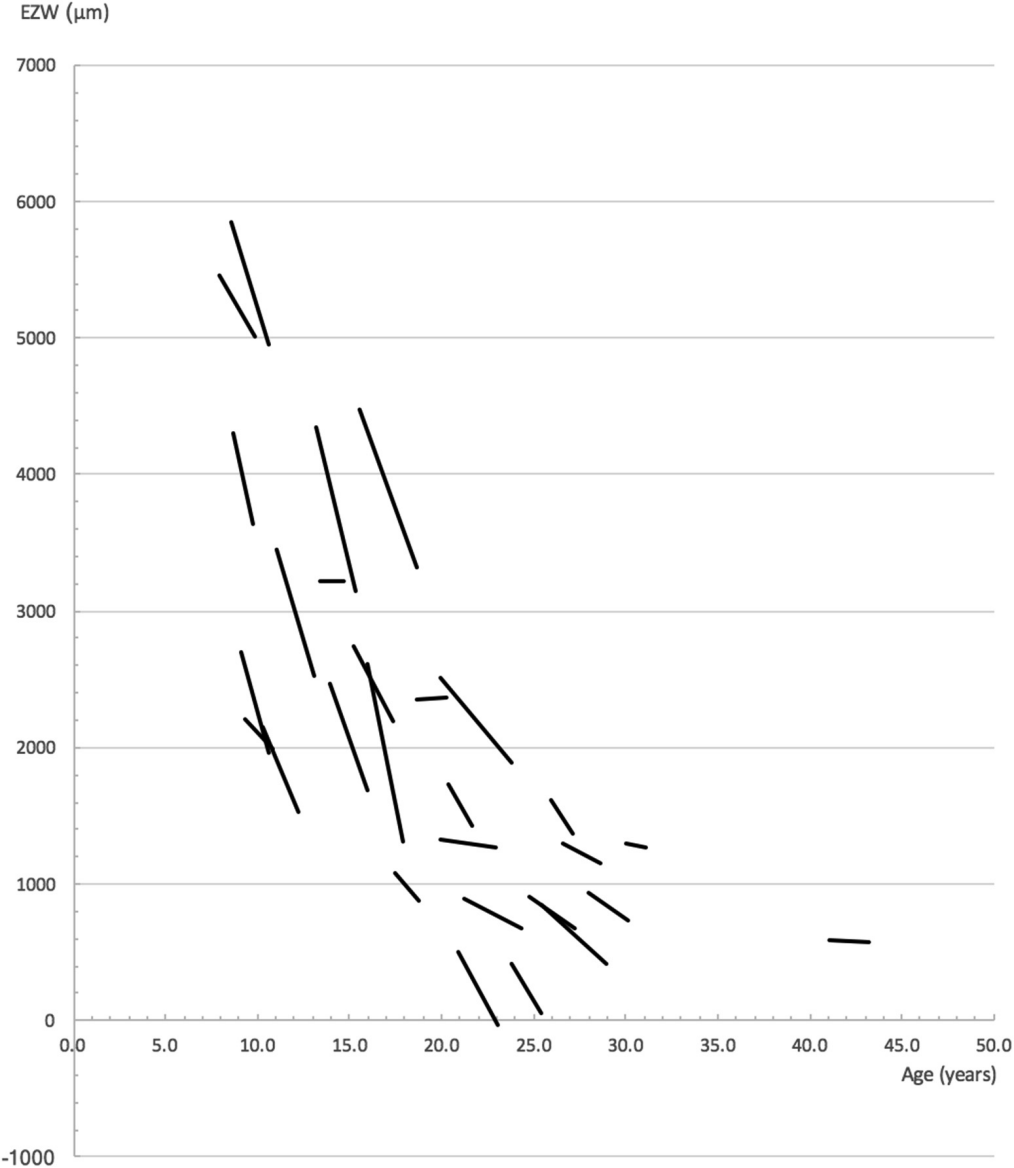
Linear trend lines indicating progression, plotted from observations of ellipzoid zone width (EZW). Each line represents the right eye of a subject. Data from 28 subjects who underwent ≥3 observations over an interval >1 year are shown.

Progression rates for individual subjects were calculated using the following method: for each eye of each subject, EZ measurements obtained with each metric were plotted as a function of age on separate scatterplots. As shown in Figure 4, linear trendlines were fitted to data points using the least squares method in Excel (version 15.41; Microsoft, Inc., Redmond, WA, USA). Progression rates for each eye of each subject were obtained from trend line gradients.

Additional statistical analyses were performed with XLSTAT software (version 2018.1; Addinsoft, New York, New York, USA). Data were inspected for normality and log transformation performed before conducting tests of statistical analyses where required. Interocular correlations at baseline and progression were investigated with the Pearson correlation coefficient (Table 3). Following this, right and left eye data were combined to investigate any correlation between age and baseline values, age and progression, and correlation between progression and baseline values (Table 3).

**Table 3:** Associations at Baseline, Progression, and With Age

Parameters	Pearson Correlation Coefficient, r	*P* Value
Interocular correlation at baseline		
EZW	0.9410	<.0001
EZA	0.9725	<.0001
Interocular correlation of progression rate		
EZW	0.6535	.0002
EZA	0.9340	<.0001
Age with baseline		
EZW	−0.6061	<.0001
EZA	−0.6381	<.0001
Age with progression rate		
EZW	−0.5751	<.0001
EZA	−0.7386	<.0001
Baseline value with progression rate		
EZW	0.4524	.0005
EZA	0.8307	<.0001

EZA = ellipzoid zone area; EZW = ellipzoid zone width.

Significance level alpha was set at 0.025 following Bonferroni correction for simultaneous analysis with 2 metrics (EZW and EZA).

The effects of age and mutation on baseline and progression were assessed with a 2-way analysis of variance (ANOVA), as shown in Table 4. Subject age was calculated from birth to time at baseline visit and was further divided into 5 categories: category 1, <10 years of age; category 2, 10 to <15 years of age; category 3, 15 to <20 years of age; category 4, 20 to <25 years of age; and category 5, ≥25 years of age. For the assessment of genotype, subjects were categorized into groups based on predicted effects of mutations: those will null allele mutations (premature stop codons or frameshifts leading to premature stop codons in exons 1-14) or those with mutations that were likely to result in translation of a variant protein product (missense mutations and mutations in ORF15). Splice-site mutations were separately grouped because of the unpredictability of their effects.^19^ In instances of a significant ANOVA result, post hoc multiple pairwise comparisons were conducted with Tukey test.

**Table 4:** Results of a 2-Way Analysis of Variance Investigating the Effects of Age and Mutation Function on Baseline Values and Progression Rates, as Characterized by EZW and EZA

	Baseline EZW (μm), Mean ± SD (No. of Eyes)	Baseline EZA (mm^2^), Mean ± SD (No. of Eyes)	EZW Rate (μm/Year), Mean ± SD (No. of Eyes)	EZA Rate (mm^2^/Year), Mean ± SD (No. of Eyes)
Age category				
1	3870.83 ± 1373.53 (12)	13.15 ± 9.06 (8)	351.52 ± 189.32 (10)	1.78 ± 1.21 (8)
2	2950.93 ± 1534.45 (14)	8.27 ± 7.74 (8)	331.65 ± 174.52 (10)	2.04 ± 0.27 (2)
3	1906.29 ± 1369.78 (14)	3.12 ± 3.22 (10)	302.76 ± 247.86 (10)	0.93 ± 0.77 (8)
4	1038.72 ± 578.26 (18)	0.89 ± 0.79 (18)	160.95 ± 98.33 (14)	0.12 ± 0.06 (12)
5	893.61 ± 453.09 (18)	0.57 ± 0.39 (18)	80.52 ± 70.10 (12)	0.08 ± 0.10 (12)
*P* value (ANOVA)	<.0001	<.0001	.0038	.0002
Tukey test	Category 1 vs categories 4 and 5 (*P* < .0001 for all); category 2 vs categories 4 and 5 (*P* < .0001); category 1 vs category 3 (*P* = .0003)	Category 1 vs categories 4 and 5 (*P* < .0001 for all); category 1 vs category 3 (*P* = .0002); category 2 vs category 4 (*P* = .0029); category 2 vs category 5 (*P* = .0017)	Category 1 vs category 5 (*P* = .0007); category 1 vs category 4 (*P* = .0224); category 2 vs category 5 (*P* = .0019); category 3 vs category 5 (*P* = .0074)	Category 1 vs categories 4 and 5 (*P* < .0001); category 2 vs category 4 (*P* = .0059); category 2 vs category 5 (*P* = .0050)
Mutation function				
Null function	1446.40 ± 775.13 (20)	1.33 ± 1.26 (18)	106.86 ± 85.83 (18)	0.17 ± 0.22 (16)
Variant protein product	2170.58 ± 1714.84 (50)	4.89 ± 7.42 (38)	287.76 ± 184.88 (34)	0.98 ± 1.12 (22)
Splice site	1962.67 ± 1747.96 (6)	3.19 ± 4.10 (6)	342.83 ± 304.20 (4)	0.98 ± 1.06 (4)
*P* value (ANOVA)	.2049	.6451	.0672	.9313
Age and mutation function interaction				
*P* value (ANOVA)	.9030	.6997	.0271	.9492

ANOVA = analysis of variance; EZA = ellipzoid zone area; EZW = ellipzoid zone width; SD = standard deviation.

Significance level alpha was set at 0.025 following Bonferroni correction. Post hoc multiple pairwise comparisons between the age categories were performed using the Tukey test with those reaching statistical significance shown. Age categories: 1 = <10 years, 2 = 10 to <15 years, 3 = 15 to <20 years, 4 = 20 to <25 years, and 5 = ≥25 years of age.

**Figure 5:**
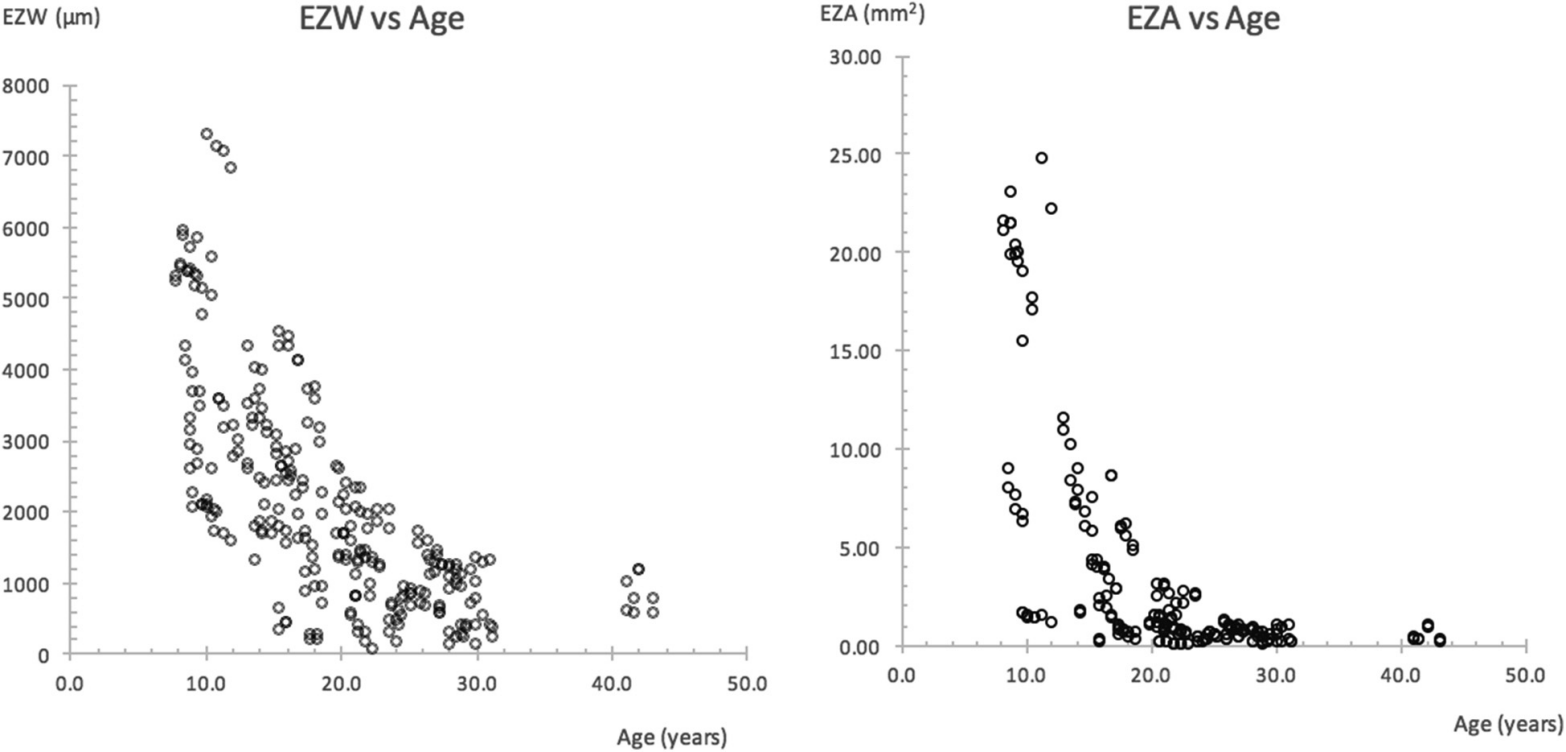
(Left) Scatterplots of ellipzoid zone width (EZW) and (right) ellipzoid zone area (EZA), both plotted against subjects' age. An exponential decline is evident for both. The reader is directed to Table 5 for further information on exponential decline rates calculated with the mixed-models method.

Overall rates of progression for both metrics were modelled using a mixed-models method (Table 5). Analysis was performed with age (calculated from birth to time of OCT image acquisition) designated as a fixed effects quantitative explanatory variable. Each eye of each subject was designated as a random effects variable. Metric values were designated as dependent variables. Values were converted into natural log form to model an exponential decline. Previous studies have shown progression to be well characterized with an exponential decay model,^20^, ^21^, ^22^ and this is further supported by evidence of exponential photoreceptor degeneration in animal models^23^ and by inspection of our data (Figure 5). Distribution of model residuals were inspected for normality.

**Table 5:** Overall Progression Modeled from EZW and EZA Data

Metric	Slope (95% CI);*P* Value	Annual % Exponential Decline Rate (95% CI)	Half-Life, Years (95% CI)
EZW	−0.0857 (−0.1024 to −0.0691); *P* < .0001	8.22 (6.67–9.73)	8.09 (6.77–10.04)
EZA	−0.1680 (−0.1974 to −0.1387); *P* < .0001	15.47 (12.95–17.91)	4.12 (3.51–5.00)

CI = confidence interval; EZA = ellipzoid zone area; EZW = ellipzoid zone width.

Annual exponential decline rates together with half-lives were calculated from slope values obtained using the mixed-models method with age designated as a fixed effects variable. All values were converted into natural log form before analyses to model an exponential decline. Half-lives with 95% CIs were calculated with the equation t_1/2_ = −log_e_ (2)/k. The significance of age exerting an effect on the model is denoted by the corresponding *P* values. Significance level alpha was set at 0.025.

The association between structure and function was investigated. EZW and EZA data as a representation of baseline structure were correlated with data obtained from tests of visual function performed at the same visit where available. Assessments of best corrected visual acuity (BCVA) were conducted with the Early Treatment Diabetic Retinopathy Study chart and contrast sensitivity (CS) assessments with the Pelli-Robson chart. BCVA was recorded in logarithm of minimum angle of resolution (logMAR) units and CS as logCS units. Automated static perimetry testing was performed on the Octopus 900 (Haag-Streit AG, Köniz, Switzerland) using a customized, radially oriented 185-point grid. Perimetry mean sensitivity (MS) values in decibel units were obtained from vendor software. The volumetric measures of V_Total_ and V_30_ in decibel-steradian units were obtained after analysis of perimetry data with third-party software (Visual Field Modeling and Analysis software). In essence, V_Total_ and V_30_ are metrics that characterize the total amount of sensitivity in the hill-of-vision as defined by the entire test grid (V_Total_) or that which is contained within a central circle of a 30° radius (V_30_). A comprehensive description was published by Tee and associates.^24^

Significance level alpha for all statistical tests was set at 0.025 after Bonferroni correction for simultaneous analysis on 2 metrics (EZW and EZA).

## Results

All 38 subjects in this study possessed bilateral EZs visible at the time of baseline imaging. Mean ± SD age for all subjects at baseline was 19.41 ± 8.32 years, ranging from 8.37 to 42.12 years. Seventeen subjects possessed mutations in exons 1-14 and 21 in ORF15. Ten were predicted null allele mutations (all harboring exon 1-14 mutations), 25 predicted variant protein products (of which 4 were exon 1-14 mutations and 21 were ORF15 mutations), and 3 were splice-site mutations. Bilateral macular OCT volume scans for en face analysis were successfully acquired at baseline for a subset of 31 subjects. The mean ± SD age for 31 subjects was 20.79 ± 8.47 years (range 8.37–42.12 years).

### Progression Analysis

For the calculation of progression rates, linear trend lines were plotted for subjects with OCT follow-up spanning a period >1 year's duration, with a minimum of 3 imaging visits. Twenty-eight subjects had bilateral rates calculated with EZW metric. The mean ± SD follow-up interval for these 28 subjects was 2.05 ± 0.72 years (range 1.10–3.81 years). Twenty-one subjects had bilateral rates calculated with EZA metric. The mean ± SD follow-up interval for these 21 subjects was 2.00 ± 0.74 years (range 1.01–3.51 years).

### Test–Retest Repeatability Analysis

A comprehensive analysis of intraobserver test–retest repeatability is presented in Table 1. For both metrics, the mean of test–retest differences was small in comparison with baseline values. For the EZW metric, the mean test–retest difference was 21.3 μm compared with a mean EZW baseline of 1963.6 μm. Likewise, for the EZA metric, the mean test–retest difference was 0.025 mm^2^ compared with a mean baseline value of 3.70 mm^2^. Calculated 95% LOA lay between −315.7 μm and 273.1 μm for EZW, between −1.06 mm^2^ to 1.01 mm^2^ for EZA, and between 0.73-0.57 mm^2^ for EZA-ART12. Test–retest variability of 15% for EZW was comparable to test–retest variability of 17% for EZA-ART12, compared with a test–retest variability of 28% for EZA.

### Baseline Values, Progression Rates, and Assessment of Interocular Symmetry

Descriptive statistics for baseline values and progression rates are provided in Table 2, together with indices of interocular symmetry. One subject (Figure 1) was excluded from symmetry analysis because the discrepancy between the right and left eyes was very large and resulted in an outlier effect, not at all in keeping with the entire cohort. Differences in mean EZW values at baseline for right eyes (1923.8 μm) and left eyes (2003.4 μm) were statistically insignificant (*P* = .6750, paired samples *t* test). Differences in mean EZA values at baseline for right eyes (3.37 mm^2^) and left eyes (4.01 mm^2^) were also statistically insignificant (*P* = .8177, paired samples *t* test).

Interocular differences in progression rates were statistically insignificant as assessed with both metrics. The mean EZW rate was 236.82 μm per year for right eyes and 230.28 μm per year for left eyes (*P* = .9479, paired samples *t* test). The mean EZA rate was 0.61 mm^2^ per year for right eyes and 0.73 mm^2^ per year for left eyes (*P* = .3346, paired samples *t* test). The equivalent annual progression rates in percentage for EZW was 13.6% for right eyes and 12.8% for left eyes. The equivalent annual progression rates in percentage for EZA was 16.0% for right eyes and 17.5% for left eyes.

A strong interocular correlation at baseline was seen with both EZW and EZA metrics (r ≥ 0.94, *P* < .0001) and for EZA-derived progression (r = 0.93, *P* < .0001), as shown in Table 3. Interocular correlation for EZW-derived progression was also strong (r = 0.65, *P* = .0002), albeit of a lesser magnitude in comparison to EZA-derived rates.

Further analysis of interocular symmetry was performed with the Bland-Altman method. Results are given in Table 2. A good level of interocular symmetry is evident with the use of both metrics to characterize baseline structure. This is reflected in RID values of 3.34% and 3.07% for EZW and EZA, respectively. The RIV, as an index of variability in interocular symmetry at baseline, was larger with EZW metrics at 31.87% compared with an RIV of 17.58% when baseline structure was characterized by the EZA metric.

A greater level of symmetry was seen with the use of EZW over EZA metric when characterizing progression. RID for EZW rates was 1.54% compared with 8.93% for EZA. There was, however, greater variability with the use of EZW over EZA. RIV was 102.60% for rates defined with EZW compared with 78.01% for rates defined with EZA metric.

### Associations of Age, Baseline, and Progression

Correlation data are shown in Table 3. All correlations were statistically significant. A strong negative association between age and baseline is evident with both metrics (r = −0.61, *P* < .0001 for EZW; r = −0.64, *P* < .0001 for EZA). A moderate to strong negative correlation was seen between age and progression (r = −0.58, *P* < .0001 for EZW; r = −0.74, *P* < .0001 for EZA). A strong positive correlation between baseline and progression was evident with EZA (r = 0.83, *P* < .0001), with a weaker positive correlation seen with the EZW metric (r = 0.45, *P* = .0005).

The effects of age and mutation on baseline values and progression rates were further interrogated with 2-way ANOVA. Results together with mean ± SD values for each category are shown in Table 4. The effect of age was significant on baseline and progression rates as determined by both EZW and EZA metrics. Post hoc pairwise comparisons revealed significant differences between subjects in age categories 1 and 2 versus age categories 4 and 5. In contrast, the effects of mutation on baseline and progression rates were insignificant.

### Mixed-Models Analyses to Determine Overall Progression

Overall rates of progression for the entire cohort was modelled using EZW and EZA data. As shown in Table 5, an annual exponential decline rate of 8.22% was obtained with EZW data. A greater exponential rate of 15.47% for the cohort was obtained with EZA data.

### Associations of Structure and Function

Correlations between structure and function at baseline are shown in Table 6. All correlations were statistically significant. EZW correlated strongly with functional metrics V_Total_, CS, and MS (r = 0.64, 0.64, and 0.63, respectively). The same functional metrics (V_Total_, CS, and MS) also showed greatest correlations with EZA (r = 0.48, 0.60, and 0.45, respectively). Correlations between EZW with V_30_ and BCVA were moderate in strength (r = 0.59 and −0.40, respectively). Correlations between EZA with V_30_ and BCVA were weaker, albeit still significant (r = 0.38 and −0.37, respectively).

**Table 6:** Associations of Structure and Function at Baseline

Parameter	EZW Pearson Correlation Coefficient, r (*P* value)	EZA Pearson Correlation Coefficient, r (*P* value)
BCVA	−0.3959 (.0004)	−0.3741 (.0027)
CS	0.6365 (<.0001)	0.5967 (<.0001)
MS	0.6279 (<.0001)	0.4508 (.0021)
V_Total_	0.6372 (<.0001)	0.4765 (.0011)
V_30_	0.5942 (<.0001)	0.3791 (.0112)

BCVA = best corrected visual acuity (recorded in logMAR units); CS = contrast sensitivity; EZA = ellipzoid zone area; EZW = ellipzoid zone width, MS = mean sensitivity.

V_Total_ and V_30_ are volumetric metrics that characterize the total amount of sensitivity in the hill-of-vision as defined by the entire test grid (V_Total_) or that contained within a central circle of 30° radius (V_30_).

Significance level alpha was set at 0.025 after Bonferroni correction for simultaneous analysis with 2 structural metrics, EZW and EZA. Associations between EZW and BCVA/CS were studied on 76 eyes of 38 subjects. Associations between EZW and MS/V_Total_/V_30_ were studied on 53 eyes of 28 subjects (28 right and 25 left eyes). Associations between EZA and BCVA/CS were studied on 62 eyes of 31 subjects. Associations between EZA and MS/V_Total_/V_30_ were studied on 44 eyes of 23 subjects (23 right and 21 left eyes).

## Discussion

Herein we describe the first protocol-driven spectral-domain OCT study to characterize EZ changes in subjects with molecularly proven *RPGR*-associated RP using both en face generated EZA and transfoveal EZW metrics.

The mean of individual progression rates in our cohort, as calculated by linear trend lines for each eye, was 234 μm per year with the EZW metric. This is comparable (albeit slightly less than) mean progression rates in XLRP reported by others using a similar metric: 248 μm,^14^ 270 μm,^15^ and 289 μm^16^ per year. However, subjects for these 3 studies were obtained from the same source (ie, the docosahexaenoic acid trial [NCT00100230]),^25^ and therefore there is likely to be a degree of subject overlap in the 3 studies. Our equivalent mean annual progression rate is 13.2% relative to baseline values. This is greater than the annual rate of 9.6% previously reported in a study with mean baseline EZW of 3410 μm.^16^ On average, subjects in our cohort possessed a smaller residual baseline EZ.

With the EZA metric, the mean of individual progression rates in our cohort, as calculated by linear trendlines for each eye, was 0.67 mm^2^ per year (16.7%/year). This is comparable to a previously reported progression rate of 0.64 mm^2^ per year (percentage rate equivalent was not provided^15^) in a study where the EZA analysis was performed via layer segmentation and the use of third-party software because acquired volume scans of 31 b-scan density were not sufficiently dense to permit the construction of en face images with vendor software.

To our knowledge, the use of en face images to quantify EZA changes in RP has been carried out by just 1 other study, albeit on subjects with autosomal recessive RP.^18^ A mean progression rate of 0.27 mm^2^ per year (13%/year) was reported; however, subjects were followed for only 1 year and the progression rate was calculated from images acquired at just 2 time points.^18^

The other studies on XLRP described above have also calculated progression with data from only 2 time points, taken an average of 2 years apart.^15^, ^16^ In these studies, only 1 eye of each subject was chosen for analysis; in the presence of multiple scans, the one with the clearest EZ band was chosen.^16^ The possibility of selection bias influencing results of these studies cannot be excluded.

We have taken a different and arguably more robust approach in our study. Data were acquired from both eyes and analyzed separately and then together. Images from multiple time points (a minimum of 3 time points) were analyzed to plot individual trend lines, from which progression rates were obtained from the slopes of trend lines. A similar approach to calculating progression rates from slopes of linear trend lines has been used by Sujirakul and associates^26^ and Cabral and associates^27^ in their analyses of EZW, with a reported mean progression rate of 140 μm per year (5.2%/year). Their cohort, however, comprised RP subjects with various inheritances (of which only 5% had XLRP) and therefore the rates are not directly comparable.

We were able to plot an exponential decline using a mixed-models approach, with data taken collectively, which afforded a wide age span. Here we found a progression rate of 8.22% per year with the EZW metric and 15.47% per year with the EZA metric. Our EZA rate, which is roughly twice the EZW progression rate, fulfils the mathematical prediction of a doubling of rate with progression tracked by area metrics (area of a circle = π [d/2]^2^). With the simultaneous use of both EZW and EZA metrics, we are able to prove this hypothesis which has been previously alluded to.^14^, ^16^

The exponential rates calculated may, in general, be more reflective of the average decline present in the population. Nonetheless, phenotypic heterogeneity is evident in this condition as demonstrated by our subjects, necessitating individual observations to be made.

### Interocular Symmetry

There is a good level of interocular symmetry at baseline, with an RID of 3% for both EZW and EZA metrics. When characterizing progression, greater interocular symmetry was found with the use of EZW over the use of EZA metrics. RID for EZW rates was 1.54% compared with 8.93% for EZA rates.

Nevertheless, there was significant variation in the degree of interocular symmetry seen across the cohort, as typified by the RIV. RIV at baseline was 17.6% and 31.9%, respectively, for EZA and EZW metrics. RIV for progression rates were 78.0 % and 102.6% for EZA and EZW, respectively.

Despite this level of variation described, interocular correlation at baseline as assessed with both metrics were strong (r ≥ 0.94, *P* < .0001). Interocular correlation for progression as characterized by EZA (r = 0.93, *P* < .0001) and EZW (r = 0.65, *P* = .0002) were also strong and significant. In addition, interocular differences at baseline and for progression rates with both metrics were statistically insignificant when tested with the paired samples *t* test. This highlights the inadequacies of using correlation analyses as a sole method for assessing interocular symmetry.

For example, the mean baseline EZW in our cohort was 1964 μm. Despite a small mean interocular difference of 64 μm, the 95% LOA reached 669 μm. The implication is that observations are necessary for all subjects before inferring the presence of good interocular symmetry. As such, one can argue that in-depth natural history studies are a requirement before treatment trials for many reasons, including this one.

### Test–Retest Variability

The 15% test–retest variability for EZW is comparable, albeit marginally smaller than the 17% test–retest variability found with EZA-ART12. In comparison, test–retest variability was 28% for EZA measurements derived from volume scans acquired without averaging. This finding indicates that both EZW and EZA-ART12 measurements are metrics with similar precision. Test–retest variability for both EZW and EZA-ART12 are equivalent to corresponding annual progression rates of 13% and 17%, respectively.

The greater precision seen with EZW and EZA-ART12 measurements is not unexpected. EZW measurements were made on transfoveal lines scans acquired with an average of 100 images each, rendering a high signal to noise ratio. This high average number of images is achievable as only 1 line scan is obtained per transfoveal image.

Following procurement of the Spectralis OCT2 module (Heidelberg Engineering), the significant reduction in acquisition time allowed high-resolution volume scans to be obtained with an average of 12 images per b-scan while maintaining the 193 b-scan density of each scan. EZA borders were more clearly visible on en face images generated from these ART12 volume scans (Figure 2), allowing for greater precision and better measurement repeatability, in contrast to EZA measurements made on images generated from earlier volume scans that were acquired without averaging.

### Test–Retest Variability Reported in Other Studies

None of the aforementioned studies assessed observer repeatability in patients with XLRP despite studying XLRP cohorts. One study selected 13 subjects with autosomal dominant RP (from 59 subjects including 26 with XLRP) with images measured twice over an unspecified time interval. Their intraobserver test–retest variability was estimated at 7.3% with a repeatability coefficient of 0.9 degrees (260 μm) and mean baseline EZW of 12.4 degrees (3584 μm). The mean annual constriction rate for their autosomal dominant RP cohort was 3.4%, indicating that their test–retest variability was twice the annual constriction rate for the tested subjects.^16^

Two related studies^14^, ^15^ assessed repeatability in image acquisition but not image measurement. In both studies, a different group of RP patients (autosomal recessive or simplex RP) to those reported were imaged twice on the same day. Another study whose cohort comprised of RP of mixed inheritances assessed intraobserver repeatability with images measured twice several weeks apart, and reported a repeatability coefficient of 233 μm with test–retest variability of 8.9%, which is almost twice that of their cohort annual constriction rate of 4.9%.^26^

### Comparisons of Test–Retest Variability With Indices of Interocular Symmetry

The mean of intraobserver test–retest difference is small compared with the mean of interocular difference for both metrics. For EZW, the mean intraobserver test–retest difference is 21.3 μm compared with 63.5 μm for the mean of interocular difference. For the EZA metric, the mean intraobserver test–retest difference is 0.03 mm^2^ compared with 0.10 mm^2^ for the mean of interocular difference. Both metrics are therefore suitable for use as structural OCT measures to quantify disease.

The 95% LOA for interocular symmetry with the EZW metric was −542 μm to 669 μm. In comparison, the corresponding 95% LOA for test–retest repeatability was −316 μm to 273 μm. Likewise, the test–retest repeatability coefficient of 294 μm for EZW is less than half its corresponding interocular coefficient of 605 μm. This finding of a test–retest repeatability that is under half that of expected interocular symmetry values further indicates that the EZW metric is reliable for use, especially where quantification of disease in the fellow eye is important—for example, in cases where the fellow eye would be expected to act as a control to the eye undergoing the treatment trial.

With regard to the EZA metric, the 95% LOA for interocular symmetry of −0.49 mm^2^ to 0.70 mm^2^ is approximately similar to the corresponding 95% LOA for test–retest repeatability of −0.73 mm^2^ to 0.57 mm^2^ obtained with EZA-ART12. The test–retest repeatability coefficient of 0.65 mm^2^ is also approximately similar to the corresponding interocular coefficient of 0.59 mm^2^. These findings again indicate that the EZA is a suitable metric for use, particularly when measurements are derived from dense volume scans obtained with good image averaging protocols.

The 95% LOA for EZA test–retest repeatability performed on scans acquired without averaging was larger at −1.06 mm^2^ to 1.01 mm^2^. As mentioned, EZA measurements were not as precise when made on en face images derived from volume scans acquired without averaging on the OCT1 during the initial period of the study. Nevertheless, these earlier EZA findings are of value and can play an important role as a secondary OCT metric to corroborate and confirm findings made with the EZW metric.

### Associations and Effects of Age on Baseline and Progression Rates

We found a strong negative correlation between age and baseline, indicating that the EZ is smaller in older eyes. The moderate to strong negative correlation found between age and progression rates, together with the strong positive correlation between baseline and progression indicates that in general, progression is greater in younger eyes possessing a larger baseline.

Results from the ANOVA (Table 4) further demonstrate the significant effects of age on baseline and progression rates. The largest baseline values and progression rates are seen in the youngest subjects of the cohort. Post hoc comparisons confirm the biggest differences in baseline and rates were between subjects in younger age categories (categories 1 and 2) compared with those in the older age categories (categories 4 and 5). In contrast, the effects of mutation on baseline and progression were insignificant. Our current findings substantiate those of our previous work.^17^, ^22^

### Correlation of Structure and Function

We have demonstrated good functional correlation with the use of both EZW and EZA as structural metrics. Correlations between EZW and functional metrics were stronger overall; however, functional correlations with EZA were also significant. These findings provide further support for the use of both structural metrics as surrogate markers of disease in *RPGR*-RP, with the demonstrated functional correlation being of key importance to both patients and regulators alike.

In conclusion, we have provided and discussed prospectively acquired spectral-domain OCT data in a cohort of subjects with *RPGR*-RP with a particular focus on the EZ as a structural biomarker of disease. The use of 2 distinct EZ metrics in conjunction adds to the robustness of this study. Both EZW and EZA metrics provide sensitive and complementary parameters to characterize structure and progression in the condition. We anticipate our natural history findings to inform recently commenced *RPGR* treatment trials, both in the recruitment of trial subjects and in adjudicating treatment responses. Our findings will also be of use to clinicians caring for patients with *RPGR*-RP and other researchers in the expanding field of phenotyping inherited retinal conditions.

## References

[1] Sharon D., Sandberg M.A., Rabe V.W., Stillberger M., Dryja T.P., Berson E.L. (2003). RP2 and RPGR mutations and clinical correlations in patients with X-linked retinitis pigmentosa. Am J Hum Genet.

[2] Shu X., Black G.C., Rice J.M. (2007). RPGR mutation analysis and disease: an update. Human Mutat.

[3] Pelletier V., Jambou M., Delphin N. (2007). Comprehensive survey of mutations in RP2 and RPGR in patients affected with distinct retinal dystrophies: genotype-phenotype correlations and impact on genetic counseling. Human Mutat.

[4] Tee J.J., Smith A.J., Hardcastle A.J., Michaelides M. (2016). RPGR-associated retinopathy: clinical features, molecular genetics, animal models and therapeutic options. Br J Ophthalmol.

[5] Schwahn U., Lenzner S., Dong J. (1998). Positional cloning of the gene for X-linked retinitis pigmentosa 2. Nat Genet.

[6] Hardcastle A.J., Thiselton D.L., Van Maldergem L. (1999). Mutations in the RP2 gene cause disease in 10% of families with familial X-linked retinitis pigmentosa assessed in this study. Am J Hum Genet.

[7] Breuer D.K., Yashar B.M., Filippova E. (2002). A comprehensive mutation analysis of RP2 and RPGR in a North American cohort of families with X-linked retinitis pigmentosa. Am J Hum Genet.

[8] Jacobson S.G., Aleman T.S., Sumaroka A. (2009). Disease boundaries in the retina of patients with Usher syndrome caused by MYO7A gene mutations. Invest Ophthalmol Vis Sci.

[9] Hood D.C., Lazow M.A., Locke K.G., Greenstein V.C., Birch D.G. (2011). The transition zone between healthy and diseased retina in patients with retinitis pigmentosa. Invest Ophthalmol Vis Sci.

[10] Aizawa S., Mitamura Y., Baba T., Hagiwara A., Ogata K., Yamamoto S. (2009). Correlation between visual function and photoreceptor inner/outer segment junction in patients with retinitis pigmentosa. Eye (Lond).

[11] Fischer M.D., Fleischhauer J.C., Gillies M.C., Sutter F.K., Helbig H., Barthelmes D. (2008). A new method to monitor visual field defects caused by photoreceptor degeneration by quantitative optical coherence tomography. Invest Ophthalmol Vis Sci.

[12] Hood D.C., Ramachandran R., Holopigian K., Lazow M., Birch D.G., Greenstein V.C. (2011). Method for deriving visual field boundaries from OCT scans of patients with retinitis pigmentosa. Biomed Opt Express.

[13] Birch D.G., Locke K.G., Felius J. (2015). Rates of decline in regions of the visual field defined by frequency-domain optical coherence tomography in patients with RPGR-mediated X-linked retinitis pigmentosa. Ophthalmology.

[14] Birch D.G., Locke K.G., Wen Y., Locke K.I., Hoffman D.R., Hood D.C. (2013). Spectral-domain optical coherence tomography measures of outer segment layer progression in patients with X-linked retinitis pigmentosa. JAMA Ophthalmol.

[15] Ramachandran R., Zhou L., Locke K.G., Birch D.G., Hood D.C. (2013). A comparison of methods for tracking progression in X-linked retinitis pigmentosa using frequency domain OCT. Transl Vis Sci Technol.

[16] Cai C.X., Locke K.G., Ramachandran R., Birch D.G., Hood D.C. (2014). A comparison of progressive loss of the ellipsoid zone (EZ) band in autosomal dominant and X-linked retinitis pigmentosa. Invest Ophthalmol Vis Sci.

[17] Tee J.J.L., Carroll J., Webster A.R., Michaelides M. (2017). Quantitative analysis of retinal structure using spectral-domain optical coherence tomography in RPGR-associated retinopathy. Am J Ophthalmol.

[18] Hariri A.H., Zhang H.Y., Ho A. (2016). Quantification of ellipsoid zone changes in retinitis pigmentosa using en face spectral domain-optical coherence tomography. JAMA Ophthalmol.

[19] Fahim A.T., Bowne S.J., Sullivan L.S. (2011). Allelic heterogeneity and genetic modifier loci contribute to clinical variation in males with X-linked retinitis pigmentosa due to RPGR mutations. PloS One.

[20] Massof R.W., Dagnelie G., Benzschawel T. (1990). First order dynamics of visual field loss in retinitis pigmentosa. Clin Vision Sci.

[21] Sandberg M.A., Rosner B., Weigel-DiFranco C., Dryja T.P., Berson E.L. (2007). Disease course of patients with X-linked retinitis pigmentosa due to RPGR gene mutations. Invest Ophthalmol Vis Sci.

[22] Tee J.J.L., Kalitzeos A., Webster A.R., Peto T., Michaelides M. (2018). Quantitative analysis of hyperautofluorescent rings to characterize the natural history and progression in RPGR-associated retinopathy. Retina.

[23] Clarke G., Collins R.A., Leavitt B.R. (2000). A one-hit model of cell death in inherited neuronal degenerations. Nature.

[24] Tee J.J.L., Yang Y., Kalitzeos A. (2018). Characterization of visual function, interocular variability and progression using static perimetry-derived metrics in RPGR-associated retinopathy. Invest Opthalmol Vis Sci.

[25] Hoffman D.R., Hughbanks-Wheaton D.K., Pearson N.S. (2014). Four-year placebo-controlled trial of docosahexaenoic acid in X-linked retinitis pigmentosa (DHAX trial): a randomized clinical trial. JAMA Ophthalmol.

[26] Sujirakul T., Lin M.K., Duong J., Wei Y., Lopez-Pintado S., Tsang S.H. (2015). Multimodal imaging of central retinal disease progression in a 2-year mean follow-up of retinitis pigmentosa. Am J Ophthalmol.

[27] Cabral T., Sengillo J.D., Duong J.K. (2017). Retrospective analysis of structural disease progression in retinitis pigmentosa utilizing multimodal imaging. Sci Rep.

